# Stiff skin, soft core: soft backings enhance the conformability and friction of fibre-reinforced adhesives

**DOI:** 10.1098/rsos.221263

**Published:** 2023-03-08

**Authors:** Niels C. Glaser, Julian K. A. Langowski

**Affiliations:** ^1^ Department of BioMechanical Engineering, Faculty of Mechanical, Maritime and Materials Engineering, Delft University of Technology, Mekelweg 2, 2628 CD Delft, The Netherlands; ^2^ Experimental Zoology Group, Department of Animal Sciences, Wageningen University and Research, De Elst 1, 6708 WD Wageningen, The Netherlands

**Keywords:** reversible adhesion, compliance control, bioadhesion, biomimetics, bioinspiration, contact mechanics

## Abstract

Biomimetic adhesives with a stiff fibre-reinforced base layer generate strong attachment, even without bioinspired micropatterning of the contact surface. However, current fibre-reinforced adhesive designs are still less versatile with respect to substrate variability than their biological counterparts. In this study, we enhance the comformability of a fibre-reinforced adhesive on curved substrates by adding bioinspired soft backings. We designed and fabricated soft backing variations (polyurethane foams and silicone hydroskeletons) with varying compressive stiffnesses that mimic the soft viscoelastic structures in the adhesive appendages of tree frogs, geckos and other animals. The backings were mounted on a smooth silicone layer enforced with a polyester mesh, and we experimentally investigated the contact area and friction performance of these adhesives on a curved substrate. The results show that the contact area and friction created by a fibre-reinforced adhesive with a soft backing in contact with a non-flat substrate scale inversely with backing stiffness. The integration of stiff fibre-reinforcement with a compressible backing represents an important step in bringing bioinspired adhesives out of the laboratory and into the real world, for example in soft robotic grippers. Moreover, our findings stimulate further research into the role of soft tissues in biological adhesive systems.

## 1. Introduction

Biological adhesive systems show fascinating features such as substrate tolerance, rapid reversibility and longevity [1]. Think, for example, of the vastly different substrates (e.g. smooth leaves, wet rocks and dry bark) a tree frog can adhere to [2–5], the high velocity at which a gecko can move across vertical and overhanging substrates [6,7], and the countless substrate contacts undergone by the adhesive tarsal pads of a fly during its life time. Bioadhesion research aims to sharpen our understanding of the complex physico-chemical mechanisms that underlie this striking performance, which is relevant to both fundamental and applied scientific research. On the one hand, insights into the functioning of bioadhesive systems contribute to an assessment of the evolutionary history of these animals [8–11]. On the other hand, the remarkable performance of bioadhesive systems has motivated the design of various biomimetic technologies, such as tree-frog-inspired adhesives [12], gecko-inspired climbing robots [13], soft robotic grippers [14] and bioinspired surgical instruments [15]. Fundamental bioadhesion research forms the indispensable first step on the way towards such biomimetic innovations.

The adhesive contact cycle between any (biological or synthetic) reversible adhesive and a substrate can be divided into three phases: (i) contact formation, in which the adhesive is pressed onto the substrate to form the largest possible contact area; (ii) contact maintenance, in which the adhesive contact needs to withstand a tensile load that is applied either perpendicularly (i.e. adhesion) or parallel (i.e. friction, elsewhere also referred to as shear adhesion (e.g. [16]) to the substrate surface, and (iii) contact release, in which the adhesive is detached from the substrate. Biological adhesive systems are adapted to function in all three phases. For example, contact formation is supported through micro- to nanoscopic fibre- or pillar-like structures on the adhesive pads of geckos, tree frogs and insects, which increase the conformability to micro- and nanorough substrates [17–19]. Subepidermal fibrous structures deeper inside the adhesive organ help to distribute tensile loads over the contact area, thus avoiding local detachment and maintaining contact [20–22]. Finally, contact is quickly and easily released by peeling off the pad from the substrate (e.g. [23]).

Discoveries of such fundamental mechanisms of bioadhesion are milestones for the design of biomimetic adhesives with an enhanced performance during one or multiple phases of contact. For example, countless prototypes of fibrillar adhesive surfaces with increased substrate conformability have been designed (e.g. [24–28]), some of which have even reached commercialization (e.g. Gecko Tape and Gecko Nanoplast). Furthermore, Bartlett *et al.* [20,29,30] developed a novel type of biomimetic adhesive that attaches strongly despite lacking any surface patterning. Inspired by the fibre-reinforced toes of geckos, they created a commercially available smooth adhesive with stiff backing (Geckskin) that maximizes attachment strength by distributing loads across the contact surface.

The attachment performance of these synthetic fibre-reinforced adhesives—and of many biological ones [31]—has been investigated mostly on flat smooth substrates such as glass plates. On such substrates, contact formation is arguably less challenging than contact maintenance, which raises the question: How well do biomimetic fibre-reinforced adhesives perform on ‘real-world’ substrates (e.g. a strawberry or tree branch) that typically show topographic irregularities ranging in size from nano- to centimetres? One can easily imagine manually pressing a fibre-reinforced adhesive into contact with a flat plate, but how would a robotic gripper bring its adhesive surface into contact with a non-flat object and maintain this contact?

**Figure 1.**
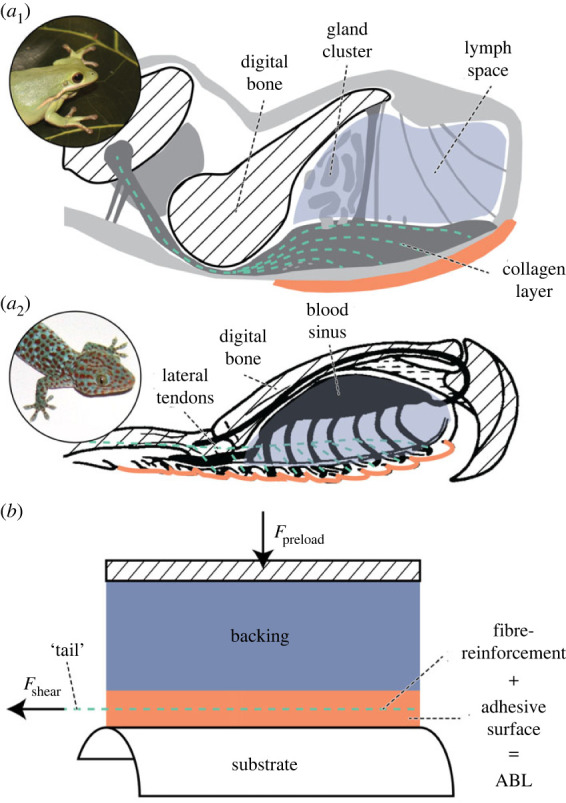
Schematic of the functional morphology of the adhesive toe tip of the North American green tree frog *Hyla cinerea* (*a*_1_; modified after [22]; inset by Julian K.A. Langowski) and the Tokay gecko *Gekko gecko* (*a*_2_; modified after [32]; inset from Wikimedia Commons, author: BacLuong; public domain) showing the micropatterened adhesive surface (orange), internal fibre-reinforcement (green dashed) and underlying soft backing (blue). (*b*) Schematic of the proposed bioinspired design of a fibre-reinforced adhesive with soft backing for enhanced conformability and friction on a non-flat substrate.

To address this question, we revisit the functional toe morphology of geckos and tree frogs. Both groups of animals possess—next to the aforementioned nano- to microscopic patterning of the adhesive toe surface, and the fibrous connection between adhesive surface and skeleton—distinct volumes of relatively soft materials between the adhesive surface and the penultimate finger bone. In various tree frog species, the volume between adhesive epidermis and finger bone (i.e. the actuated structure) contains fluid-filled structures (a cluster of mucus glands, a lymph space and a dense dermal network of capillary blood vessels; figure 1*a*_1_; [22,33–37]). The resulting low compressive stiffness of tree frog toe pads has been hypothesized to enable conformation to non-flat substrates [38]. Similarly, Russell [39] described in the toes of the Tokay gecko (*Gekko gecko*) a complex network of blood vessels, including a venous blood sinus (figure 1*a*_2_) between the adhesive epidermis and the finger bone, which may act as a highly conformable hydroskeleton with variable stiffness [40,41].

Here, we take inspiration from the functional morphology of gecko and tree frog toes, and propose a novel design paradigm for smooth fibre-reinforced adhesives with a soft backing that enables conformation and strong attachment to non-flat substrates. We manufacture different adhesive variations with variable backing stiffness and measure the effect thereof on friction performance. Overall, we show that the biomimetic combination of a high stiffness in shear loading with a low compressive stiffness results in a versatile adhesive that performs well on non-flat substrates. This proof-of-concept emphasizes the need for an integrative approach in biomimetic design, and provides quantitative support for the so-far untested hypotheses on the role of soft backings in biological adhesives.

## 2. Methods

### 2.1. Adhesive design and fabrication

Taking inspiration from the toes of tree frogs and geckos, we designed an adhesive that comprises three key functional components: (i) a tacky surface layer that can conform to micro- to nanoscopic substrate roughness features (henceforth ‘adhesive surface’), analogously to the micropatterned surface of the adhesive pads of geckos and tree frogs; (ii) a fibre-reinforced base of the adhesive surface that is stiff in shear loading (henceforth ‘fibre-reinforcement’; we refer to the free end of the fibre-reinforcement as ‘tail’); and (iii) a soft ‘backing’ below the adhesive surface and fibre-reinforcement that enables conformation to macroscopic substrate roughness features. We refer to the combination of adhesive surface and fibre-reinforcement as ‘adhesive base layer’ (ABL), and to the entire system as ‘adhesive’.

To investigate the effect of the novel backing element on the adhesive’s friction performance on a non-flat substrate, we kept the ABL constant while varying the backing design and its mechanical properties. With the friction force $F_{∥}$ being the product of maximum shear stress $\sigma_{∥}$ and contact area *A*, and contact area depending on the adhesive’s compressibility, we expect that the adhesive’s friction scales inversely with its compressive stiffness on a non-flat substrate. Due to COVID-19 restrictions, all adhesive manufacturing and experimentation were conducted in a home-office setting using custom-made procedures and setups.

#### 2.1.1. Adhesive base layer

The ABL is designed following Bartlett *et al.* [42], and consists of a soft silicone matrix that is internally reinforced with a polyester mesh. To produce the ABL, we clamped rectangular midge mesh (30 fibres in tail direction and 18 fibres in width direction per square inch) between two 0.5 mm thick polystyrene plates with 5 cm × 5 cm large rectangular cut-outs, which were placed on a smooth glass plate. Gaps between glass, mesh and polystyrene were sealed with petroleum jelly. We prepared silicone rubber (Resion rubber SR1, shore hardness 8A, polyestershoppen.nl) at a part A to B weight ratio of 1 : 5, coloured the silicone with white pigment (1 wt% white silicone pigment, siliconesandmore.nl) for later contact area imaging, and filled the cut-outs with silicone. Air pockets were removed manually if present. After curing, the mesh-reinforced silicone was removed from the polystyrene plates and glass, cut into 5 cm × 5 cm large adhesives with free mesh tails, and cleaned with isopropanol and water. The silicone surface that cured onto the glass served as contact surface in the later experiments.

To assess variability of the manufactured ABL specimen, we measured their friction performance on a glass cylinder, whose long axis runs parallel to the ABL’s tail (see §2.2 for setup details). When reusing a single specimen for 30 consecutive repetitions, static and dynamic friction were 46.0±1.3 N and 33.3±1.6 N, respectively (mean ± s.d.), corresponding to an intra-specimen variability below 4.3%. Additionally, we measured all 26 ABL specimens used in the later experiments to test for inter-specimen variability. With a static and dynamic friction of 53.3±5.5 N and 33.4±1.1 N, variability between specimens was below 10.4%.

#### 2.1.2. Backings

**Figure 2.**
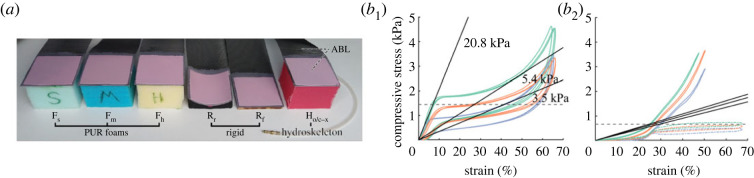
(*a*) Photographs of manufactured backing variations (PUR-foams, rigid controls, hydroskeleton) glued onto adhesive base layer (ABL). (*b*) Compressive stress–strain diagrams of PUR-foam and hydroskeleton backings. (*b*_1_) Stress–strain curves of foam backings (solid coloured curves) of differing nominal relative stiffness gradings (soft—blue, medium—orange, hard—green). This stiffness variation is confirmed also by secant compressive moduli extracted from the stress–strain curves (secant modulus = slope of the secant drawn between the origin and the intersection of stress–strain curve and average compressive stress level applied in the later friction trials; grey dashed line). (*b*_2_) Stress–strain curves of open (solid coloured lines) and closed (dashed-dotted coloured lines) hydroskeleton backings with varying internal pressure (0.1 kPa—blue, 0.2 kPa—orange, 0.3 kPa—green). For the closed backings, a positive scaling of secant modulus with increasing internal pressure (2.3 kPa @ 0.1 kPa, 2.5 kPa @ 0.2 kPa, 2.7 kPa @ 0.3 kPa) was extracted as described above.

Polyurethane (PUR) foams and silicone hydroskeletons were used to mimic the soft materials found in gecko and tree frog toes (figure 2*a* and table 1). All backings had a size of 5 cm × 5 cm × 3.5 cm (width × length × height), such that they fit onto the ABL patches. Backing height was chosen equal to the radius of curvature of the used cylindrical substrate, such that a backing would form full contact with the substrate under full compression. Backing width (i.e. length of the backing wrapped around the curved substrate) was set smaller than the cylinder diameter as extreme deformations and conformation at the outer contact regions were unexpected, and backing length was chosen equal to its width. Rigid backings served as reference. The different backings were bonded to the ABL with silicone (Resion rubber SR1, part A to B weight ratio of 1 : 1).

**Table 1.** Overview of backing layer variations and experimental settings.

backing type	variation	*N*_specimen_ : *N*_trial_	*F*_pre_ (N)
polyurethane foam	F_s_	5 : 3	3.6
	F_m_		
	F_h_		
silicone hydroskeleton^a^	H_c_-1	1 : 5	1.7
	H_c_-2		
	H_c_-3		
	H_o_-1		
	H_o_-2		
	H_o_-3		
rigid references	R_f_	5 : 3	3.6
	R_r_		

^a^For all hydroskeleton variations the same specimen was used.

We confirmed qualitative differences in mechanical properties of the different manufactured backings using a custom-made compression setup. Each fabricated backing material was placed between a stationary plate and a moving plate that was mounted via a force sensor to a linear actuator, which allowed measuring compressive stress–strain curves while moving the two plates towards each other. The non-stationary plate moved at 0.5 mm s^−1^, started at an initial height, compressed the backings for a defined distance and returned to zero displacement. For the PUR-foams, the compressive stress at a given strain differed between the three different foam backings, suggesting relative differences in compressive stiffness (figure 2*b*). Similarly, compressive stress increased for the hydroskeleton variations with applied internal pressure. Stresses were systematically higher for closed hydroskeletons as compared to open ones.

**PUR-foams** PUR-based memory foam combines two relevant mechanical properties of the soft materials found in the toe pads of tree frogs and geckos: (i) PUR-foam has a relatively low compressive modulus in the order of kilopascal to megapascal [43–45], similar to the bulk compressive modulus of biological adhesives [38,46]. (ii) The fluid-filled structures inside frog and gecko pads may be abstracted as an open-celled sponge, which allows movement of a contained fluid and thus is viscoelastic.

We cut PUR-blocks with the specified dimensions manually from three different commercially available foam sheets that varied in relative stiffness (figure 2*b*_1_): soft (henceforth referred to as ‘F_s_’; Memory foam SG 65 Sensus, schuimrubbergigant.nl), medium (‘F_m_’; Memory foam SG 57, schuimrubberbetaalbaar.nl) and hard (‘F_h_’; Memory foam SG 50, schuimwinkel.nl).

**Silicone hydroskeletons** The fluid-filled space inside the adhesive pads of tree frogs (i.e. mucus glands, lymph space and capillary blood vessel network) and geckos (i.e. venous blood sinus) may also be abstracted as a volume of fluid inside an elastic container. In line with the hypotheses by Russell [39] and Barnes *et al.* [38], such a structure effectively acts as a hydrostatic skeleton (i.e. hydroskeleton) with a relatively low stiffness in compression and tension.

We modelled this hypothesized functionality in two variations of synthetic hydroskeletons: (i) we created a ‘closed hydroskeleton’ consisting of a compliant hull containing a constant volume of air (henceforth referred to as ‘H_c-x_’, where ‘x’ stands for the applied pressure of 0.1, 0.2 and 0.3 kPa). (ii) We opened the closed hydroskeleton and connected it to a larger reservoir volume to mimic the liquid-filled spaces in tree frog and gecko toes (blood vessels and lymph space) that are potentially connected to a larger fluidic system (i.e. circulatory or lymphatic system, respectively). Air could leave from the hull to the reservoir at a specified pressure, thus enabling constant internal pressure of this so-called ‘open hydroskeleton’ (‘H_o-x_’). Both variations have a tunable initial internal pressure to vary compressive stiffness.

For both hydroskeleton variations, the hull had a size of 5 cm × 5 cm × 3.5 cm and a wall thickness of 1 mm. Silicone was prepared as described above (at a 1 : 1 weight ratio), degassed in a custom-made vacuum chamber to remove bubbles and poured into a custom-made multi-part mould of the hull. The cured hull was removed from the mould and its open side was closed with a 1 mm thick silicone slab. A silicone tube (${\text{Ø}}_{\mathrm{in}}=2\;\mathrm{mm}$, ${\text{Ø}}_{\mathrm{out}}=3\;\mathrm{mm}$) was glued into an opening in one of the hull walls. Via this tube the closed hydroskeleton was serially connected to a custom-made water-column pressure sensor and a hand pump, which allowed control of the air pressure inside the hull. After reaching the desired pressure, the pressurized hull was decoupled from the rest of the system with a clamp.

The open hydroskeleton, in which air could leave the hull while maintaining constant pressure, was built similarly. A fluid reservoir of 50 L was created by connecting an inflated garbage bag (Komo; Albert Heijn) to the tube between hull and pressure sensor. With a ca. 570-fold larger volume of the reservoir compared to the hull, the air in the reservoir is barely compressed when the hull is totally compressed while maintaining nearly constant pressure (Δ*p* ≈ 0.15 kPa).

**Rigid references** The effects of the different soft backings on the conformability and friction performance of the ABL were compared against the effects of rigid backings. A 3 mm-thick plywood plate of 5 cm × 5 cm was used as rigid flat backing, which is henceforth referred to as ‘R_f_’.

A rigid curved backing (‘R_r_’) for an adhesive with the same curvature as the used substrate was made by pouring polyester (Resion polyester giethars), which was prepared with 2 wt% hardener and 2 wt% black pigment (Resion polyester pigmentpasta black), onto the top side of an adhesive layer placed on the later used cylindrical glass substrate.

### 2.2. Contact area and friction measurements

**Figure 3.**
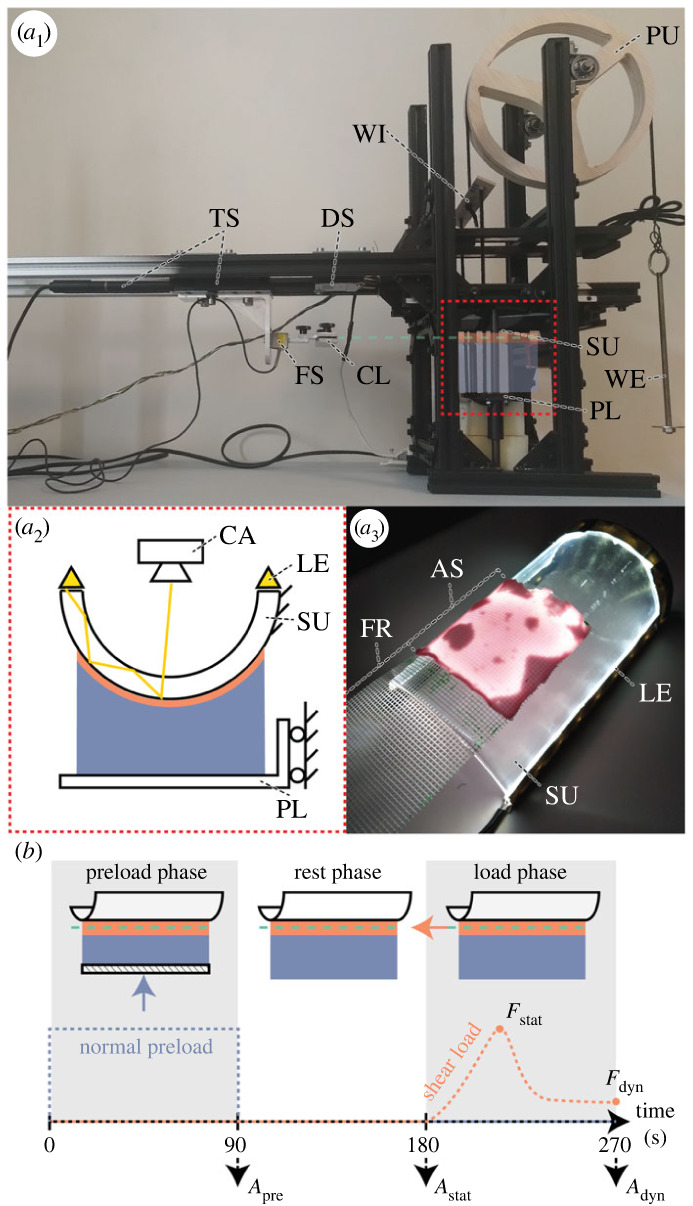
Experimental (*a*) setup and (*b*) protocol to measure the contact area and friction performance of fibre-reinforced adhesives with soft backing on a curved substrate. (*a*_1_) Overview of the experimental setup including substrate, preload, shear-load and imaging system. The position of the adhesive (semi-transparent) in the centre section (red box) of the setup is indicated schematically. For detailed description see main text. (*a*_2_) Schematic of the centre section showing the adhesive under compressive preload, and contact area imaging by frustrated total internal reflection (FTIR). (*a*_3_) FTIR-based visualization of the effective contact area (illuminated regions of the adhesive-substrate interface) of an adhesive base layer specimen in contact with the curved substrate. AS, adhesive surface; CA, camera; CL, clamp of adhesive tail; DS, distance sensor; FR, fibre-reinforcement; LE, LED strip; FS, force sensor; PL, platform; PU, pulley; SU, substrate; TS, translation stage and motor pen; WE, weight; WT, whippletree. (*b*) Schematic of the experimental protocol throughout preload, rest and load phase.

The conformability and friction performance of the novel fibre-reinforced adhesives with soft backing was assessed using a custom-made setup consisting of four components: (i) a curved substrate, (ii) a preload system to press the adhesive into contact with the substrate, (iii) a shear-load system to measure the friction performance of the adhesive during subsequent shear loading, and (iv) an imaging system to simultaneously measure the contact area (figure 3).

The substrate was a half hollow glass cylinder with an outer diameter of 7 cm, its long axis oriented horizontally, and its convex side facing downwards. The adhesive was placed with its contact surface facing upwards on a platform below the substrate. The platform could move vertically along two guiding rods, and was connected via two cables to a whippletree, which in turn was connected to a cable running over a pulley. Applying a weight to the other end of this cable allowed pressing the adhesive onto the substrate at a defined normal preload. For the PUR-foam (and rigid) backings, we applied a preload of 3.6 N, which corresponds to approximately full contact between the softest foam (F_s_) and the substrate, and for the hydroskeletons a preload of 1.7 N, which prevented the hydroskeleton from getting completely compressed.

To apply a shear load on the adhesive in contact with the substrate, the adhesive’s tail was oriented parallel to the long axis of the cylindrical substrate and clamped at a distance of 13 cm from the adhesive between two metal plates covered with double-sided tape. The clamp was connected to a load cell (LSB200 FSH00105, Futek) in series with an electrical signal amplifier (CPJ, Scaime) and an AD-converter (USB-6008, National Instruments). The connection between clamp and load cell allowed rotation in the tail plane, thus aligning the tail with the applied shear load. The force sensor was connected to a linear actuator (Z825B, Thorlabs), which moved parallel to the long axis of the cylindrical substrate over a distance of 9 mm at a velocity of 0.1 mm s^−1^. Actuator displacement was measured with a distance sensor (optoNCDT 1300-ILD1300-50, Micro Epsilon) connected to the aforementioned AD-converter. Both shear force and displacement were measured synchronously at 10 Hz.

The effective contact area between adhesive and substrate was visualized through frustrated total internal reflection (FTIR; [47,48]). A LED strip (MLS-FA-PW-12-IP20, hetledhuis.nl) was attached along the sides of the glass substrate and emitted light into the substrate, which was totally reflected within the glass. However, at locations of contact between glass and a medium with a refraction index lower than that of glass (e.g. the adhesive), light scattered and was recorded with a smartphone camera (Nokia 7.1, Daylight ISO100, 1/500 shutter; effective area resolution of 0.0042 mm^2^ per pixel).

Before each trial, the substrate was cleaned with isopropanol. Subsequently, the adhesive was preloaded for 90 s (i.e. preload phase), and the contact area (*A*_pre_) was imaged. Afterwards, the preload was removed and after another 90 s (i.e. rest phase), contact area was imaged again (*A*_stat_). In the final load phase, the adhesive was put under shear load as aforementioned, and a third image of the contact area (*A*_dyn_) was taken 1–2 s before the actuator stopped. During the load phase we also measured the load–displacement curve for each adhesive specimen. These curves showed a typical monotonic increase in shear load withstood by the adhesive up to a maximum (i.e. static friction *F*_stat_), and a subsequent drop of the withstood shear load to a lower plateau when the adhesive is sliding (i.e. dynamic friction *F*_dyn_).

*Experimental design and data analysis* The adhesives with foam and hydroskeleton backings differed in their structural design (homogeneous foam versus liquid-filled container), mechanical properties (figure 2*b*) and applied preload. Therefore, we separately examined the effects of backing stiffness variations on the conformability and friction of the fibre-reinforced adhesives for both backing types. We tested for differences in effective contact area and friction between the adhesives with foam-based backings (F_s,m,h_) as well as rigid backings (R_r,f_). For each backing variation, five adhesive specimens were tested in a randomized order, with three trials per specimen to compensate for intra-specimen variability of the ABL. Similarly, variation in conformability and friction of a single adhesive specimen with open (H_o-0.1,0.2,0.3_) and closed (H_c-0.1,0.2,0.3_) hydroskeleton backing was tested with five trials per setting in a randomized order.

The force-distance curves and contact area images were analysed using Matlab (R2018b). Distance data were smoothed using moving average filtering (window width=10 data points), and force-distance curves were aligned with respect to each other using a force threshold of 0.2 N. Static friction *F*_stat_ was chosen as the maximum friction value, and dynamic friction *F*_dyn_ was extracted 0.1 mm before the end of the sliding movement. Contact areas were quantified using serial local weighted mean transformation (‘fitgeotrans’), median filtering for noise removal using a 3 x 3 pixel neighbourhood (‘medfilt2’), image binarisation using Otsu’s method [49] (‘imbinarize’), and calibration.

Statistical analyses were performed in R (v. 4.2.2). We could not verify homoscedasticity for all forces and contact areas measured for the different adhesives using Levene’s and Bartlett’s test (see electronic supplementary material, table S1). Therefore, we used Welch’s ANOVA (‘welch_anova_test’), which does not require homogeneous variances, to test for an effect of backing variation on contact area and friction, with separate tests for the preload, rest and sliding phase. Per phase, differences between the backing variations were resolved through multiple comparison using the Games–Howell method, which also does not require homogeneous variances (5% confidence level).

While friction of stiff systems is considered independent of the apparent contact area according to Amontons’ laws of friction, friction in fact does scale with the effective contact area *A* according to Bowden and Tabor’s theory [50,51]. We measured the effective contact area of the used tacky adhesives through FTIR-based contact area measurements, and tested for the expected relation between friction and contact area by fitting robust linear regression models separately for the static (*F*_stat_ and *A*_stat_) and dynamic (*F*_dyn_ and *A*_dyn_) situation using the ‘fitlm’ function with Tukey’s bisquare method in Matlab.

## 3. Results

### 3.1. Polyurethane foam backings

**Figure 4.**
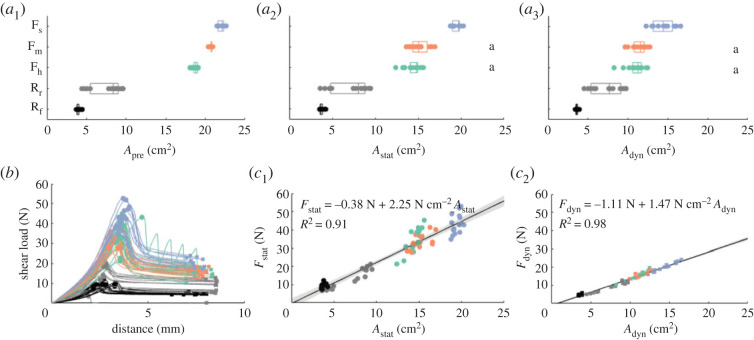
Conformability and friction performance of fibre-reinforced adhesives with soft foam backings on a curved substrate. (*a*) Effect of backing stiffness on the adhesive-substrate contact area for polyurethane foam backings with low (blue; F_s_), moderate (orange; F_m_) and high (green; F_h_) relative compressive stiffness, and with rigid flat (black; R_f_) and curved (grey; F_r_) backings during the preload (*a*_1_), rest (*a*_2_) and load (*a*_3_) phase. Small letters indicate all groupings of adhesive variations that do not differ significantly in contact area (see main text). (*b*) Shear load–distance diagram for adhesives with varying backings indicating maximum (dots; *F*_stat_) and dynamic (squares; *F*_dyn_) friction. (*c*) Relation between friction and contact area for adhesives with varying backings during rest (*c*_1_) and load (*c*_2_) phase. Equations describe the best linear fit function (black line) between friction and contact area with according coefficients of determination. 95% CIs are indicated as transparent grey patches.

The effective contact area of the adhesives with PUR-foam and rigid backings, which were preloaded with the same force, varied from 3.61 ± 0.17 cm^2^ (mean ± s.d.; R_f_ in slide phase) to 22.01 ± 0.40 cm^2^ (F_s_ in preload phase; figure 4), which corresponds to 14.5–88.0% of the maximally available surface area of 25 cm^2^. The contact area of the adhesives with rigid backings was lower than that of the adhesives with foam backing in all three phases. Among the adhesives with rigid backings, R_f_ had a lower contact area than R_r_ in all three phases. Among the adhesives with foam backings, contact area generally scaled inversely with relative foam stiffness. We observed for most adhesives a decrease in contact area throughout the trial (i.e. from rest to load phase), which was more prominent for the adhesives with foam backing if compared to the adhesives with rigid backings. Only for R_r_, contact area increased slightly during sliding.

The difference in contact area between adhesives with different foam (F_s,m,h_) and rigid (R_r,f_) backings was significant for each phase of contact (i.e. preload, rest, load), as revealed by Welch’s ANOVAs (*A*_pre_: *F*_4,31.79_ = 13673, *p* < 2.2 × 10^−16^; *A*_stat_: *F*_4,29.96_ = 3354.1, *p* < 2.2 × 10^−16^; *A*_dyn_: *F*_4,27.99_ = 585.7, *p* < 2.2 × 10^−16^). Multiple comparison with the Games–Howell test showed per contact phase significant differences in contact area between all adhesive variations, except for F_m_ and F_h_, which did not differ significantly from each other during the rest (*p* = 0.31; figure 4*a*_2_) and load phase (*p* = 0.82; figure 4*a*_3_ and electronic supplementary material, table S2).

The friction between these adhesives and the curved substrate varied over one order of magnitude from 4.70 ± 0.44 N (*F*_dyn_ of R_f_) to 43.27±5.49 N (*F*_stat_ of F_s_; figure 4*b*). A Welch’s ANOVA showed that static friction *F*_stat_ differed significantly among adhesives with different foam and rigid backings (*F*_4,30.05_ = 263.85, *p* < 2.20 × 10^−16^; electronic supplementary material, figure S2*a*_1_). Multiple comparison using the Games–Howell test (electronic supplementary material, table S2) shows that the adhesives with rigid backings create significantly lower static friction than the ones with foam backings (*p* = 1.12 × 10^−13^ − 9.73 × 10^−9^). F_m_ and F_h_ did not differ from each other in created friction (*p* = 0.96), but created less friction than F_s_ (*p* = 7.73 × 10^−5^ − 1.17 × 10^−2^).

Dynamic friction also differed significantly between the different adhesives, as revealed by Welch’s ANOVA (*F*_4,28.7_ = 309.32, *p* < 2.20 × 10^−16^). Similarly to static friction, the adhesives with rigid backings created significantly lower friction than the ones with foam backings (*p* = 4.69 × 10^−13^ − 3.42 × 10^−6^; electronic supplementary material, figure S2*a*_2_ and table S2). Also for dynamic friction, F_m_ and F_h_ did not differ from each other in created friction (*p* = 0.95), and created less friction than F_s_ (*p* = 2.45^−4^ − 2.69 × 10^−4^). By contrast to static friction, however, the adhesive with flat rigid backing also created significantly lower friction than the one with rigid curved backing (*p* = 8.22 × 10^−4^).

Friction scaled positively with the effective contact area, across all tested adhesive variations in both the rest and slide phase as shown by linear regression (figure 4*c*). The regression between static friction *F*_stat_ and contact area during the rest phase *A*_stat_ was statistically significant (adjusted *R*^2^ = 0.914, *F*_73,71_ = 763, *p* = 1.03 × 10^−39^), with an average static frictional stress (i.e. static friction per unit contact area) of $2.25\pm 0.08\;{N\;\mathrm{cm}}^{-2}$ (estimate ± standard error; *t* = 27.62, *p* = 1.04 × 10^−39^). Also the regression between dynamic friction *F*_dyn_ and contact area during the rest phase *A*_dyn_ was statistically significant (adjusted *R*^2^ = 0.982, *F*_73,71_ = 3.88 × 10^3^, *p* = 1.08 × 10^−63^), with an average dynamic frictional stress of $1.47\pm 0.02\;{N\;\mathrm{cm}}^{-2}$ (*t* = 62.25, *p* = 1.08 × 10^−63^).

### 3.2. Hydroskeleton backings

**Figure 5.**
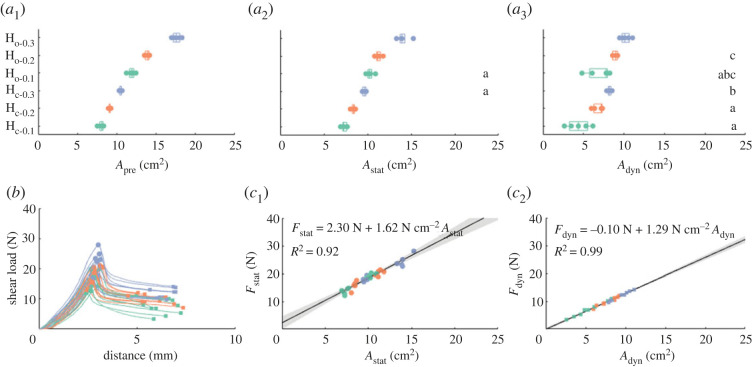
Conformability and friction performance of fibre-reinforced adhesives with soft hydroskeleton backings on a curved substrate. (*a*) Effect of backing stiffness on the adhesive-substrate contact area for open and closed hydroskeleton backings with varying internal pressure during the preload (*a*_1_), rest (*a*_2_) and load (*a*_3_) phase. Small letters indicate all groupings of adhesive variations that do not differ significantly in contact area according to Welch’s ANOVA and multiple comparison (see main text and electronic supplementary material, table S2). (*b*) Load–distance diagram for adhesives with varying backings indicating maximum (dots; *F*_stat_) and dynamic (squares; *F*_dyn_) friction. (*c*) Relation between friction and contact area for adhesives with varying backings for rest (*c*_1_), and load (*c*_2_) phase. Equations describe the best linear fit function (black line) between friction and contact area with according coefficients of determination. 95% CIs are indicated as transparent grey patches.

For the adhesives with inflatable backings, effective contact area varied from 4.39 ± 1.40 cm^2^ (H_c-0.3_ in slide phase) to 17.59 ± 0.53 cm^2^ (H_o-0.1_ in preload phase; figure 5*a*), which corresponds to 17.6–70.4% of the total surface area of the adhesives. We generally observed for the adhesives with an open hydroskeleton backing (H_o-x_) a larger contact area than for the adhesives with closed hydroskeleton backing (H_c-x_). Moreover, contact area scaled inversely with the internal pressure of the hydroskeleton backings. As for the foam-based backings, contact area decreased throughout the trial (i.e. from rest to load phase).

Within each contact phase (i.e. preload, rest, load), contact area variation between adhesives with open and closed hydroskeleton backings as well as different internal pressures was significant according to Welch’s ANOVAs (*A*_pre_: *F*_5,10.77_ = 410.9, *p* = 6.5 × 10^−12^; *A*_stat_: *F*_4,11.00_ = 112.1, *p* = 4.7 × 10^−9^; *A*_dyn_: *F*_4,10.72_ = 21.9, *p* = 2.7 × 10^−5^). Multiple comparison revealed significant differences in contact area between all adhesive variations during the preload phase (figure 5*a*_1_; electronic supplementary material, table S2). During the rest phase, all adhesive variations differed from each other, except for H_c-0.1_ and H_o-0.3_ (*p* = 0.12; figure 5*a*_2_). During the load phase, differences between the adhesive variations are less clear (figure 5*a*_3_; electronic supplementary material, table S2). H_o-0.1_ has a significantly higher contact area than the other adhesive variations, which show clustering in groups with significantly differing contact areas.

The friction of the adhesives with hydroskeleton backings on a curved substrate varied from 5.60±1.90 N (*F*_dyn_ of H_c-0.3_) to 24.49±2.21 N (*F*_stat_ of H_o-0.1_; figure 5*b*). Static friction *F*_stat_ differed significantly among adhesives with different hydroskeleton backings and varying internal pressures, as shown by Welch’s ANOVA (*F*_5,11.05_ = 25.69, *p* = 9.79 × 10^−6^). Multiple comparison using the Games–Howell test shows significant differences in static friction between several groups of adhesives (electronic supplementary material, figure S2*b*_1_ and table S2). These groupings follow the general trend of adhesives with open hydroskeleton backing and with lower internal pressure creating stronger friction.

Dynamic friction also differed significantly between the different adhesives, as revealed by Welch’s ANOVA (*F*_5,10.59_ = 19.47, *p* = 5.07 × 10^−5^). Similarly to static friction, multiple comparison shows that adhesives with open hydroskeleton backing and with lower internal pressure create stronger friction (electronic supplementary material, figure S2*b*_2_ and table S2).

Friction scaled positively with the effective contact area, across all tested adhesive variations in both the rest and slide phase as shown by linear regression (figure 5*c*). The regression between static friction *F*_stat_ and contact area during the rest phase *A*_stat_ was statistically significant (adjusted *R*^2^ = 0.92, *F*_30,28_ = 344, *p* = 2.9 × 10^−17^). On average, static frictional stress was 1.62 ± 0.09 N cm^−2^ (*t* = 18.54, *p* = 2.95 × 10^−17^). Also the regression between dynamic friction *F*_dyn_ and contact area during the rest phase *A*_dyn_ was statistically significant (adjusted *R*^2^ = 0.99, *F*_30,28_ = 2.25 × 10^3^, *p* = 2.77 × 10^−28^), with an average dynamic frictional stress of 1.29 ± 0.03 N cm^−2^ (*t* = 47.39, *p* = 2.77 × 10^−28^).

## 4. Discussion and conclusion

### 4.1. Friction of bioinspired adhesives with soft backings on non-flat substrates

This study shows that the friction between an adhesive and a curved substrate scales positively with the adhesive’s ability to conform to the substrate. Among the tested adhesives, the bioinspired ones with softer backings formed larger contact areas than stiffer adhesive variations throughout the whole contact cycle when pressed onto a cylindrical glass substrate (figures 4 and 5). Moreover, both static and dynamic friction correlated linearly with respective contact areas for all tested adhesive variations. In line with our initial expectation, the adhesives with backings with lower compressive moduli were consistently found at the high end of the resulting trendlines, and vice versa.

The relation between contact area *A* and effective elastic modulus E* (i.e. a correlate of compressive modulus) can be computed analytically using Hertz theory [50] for the contact between a rigid cylinder (i.e. the substrate) and an elastic halfspace (i.e. the adhesive; see appendix for model description) as

4.1 $$A=2LR\arccos\left(1-\frac{4F}{\pi E^{*}LR}\right),$$

with adhesive length *L*, cylinder radius *R* and applied compressive load *F*. Keeping the other variables constant as in this study, this model predicts $A\propto \arccos\left(1-{E^{*}}^{-1}\right)$, which approximates for the specific values *L* = 0.05 m, *R* = 0.035 m and *F* = 3.6 N over the range E* = 10 − 10 × 10^7^ kPa to *A* ∝ E^*−0.5^. While we cannot directly apply this model to our data because of the unknown effective elastic moduli of the different adhesives, the predicted negative scaling of contact area with increasing adhesive stiffness qualitatively agrees with the observed trends.

A low compressive stiffness and the resulting large contact area are not the sole determinants of the friction performance of an adhesive (e.g. [52]). Bartlett *et al.* [20] proposed a general adhesion scaling law (GASL) based on energy balance to predict the attachment performance of reversible adhesive systems of arbitrary shape and material. For an adhesive with contact area *A* and compliance *C* (i.e. inverse of the stiffness *K*) in the direction of the applied tensile load, the critical force *F*_*c*_ withstood by the adhesive directly before contact release (e.g. static friction) scales as

**Figure 6.**
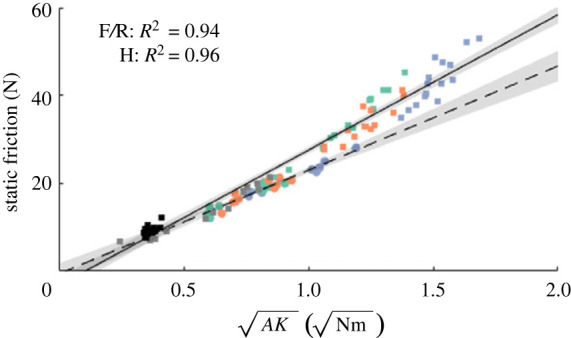
Scaling of the static friction of the tested bioinspired adhesives with soft PUR-foam and rigid backings (squares) and hydroskeleton backings (dots; for symbol explanations see figures 4 and 5) with the square-root of the product of effective contact area *A* and stiffness *K* according to the general adhesion scaling law by Bartlett *et al.* [20]. Both for the adhesives with soft PUR-foam and rigid backings (solid line; *F*_30,28_ = 438, *p* = 1.2 × 10^−18^) and the adhesives with hydroskeleton backings (dashed line; *F*_73,71_ = 1.9 × 10^3^, *p* = 5.0 × 10^−53^), we found significant linear correlations between *F* and $\sqrt{AK}$, with the slopes of the curves equal to the square roots of the average critical strain energy release rates (F/R: $G_{c}=30.8\pm 0.7\;N^{0.5}\;m^{-0.5}$, *t* = 43.65, *p* = 5.0 × 10^−53^; H: *G*_*c*_ = 23.7 ± 1.1 N^0.5^ m^−0.5^, *t* = 20.93, *p* = 1.2 × 10^−18^). 95% CIs are indicated as transparent grey patches. Stiffness values were obtained by measuring the slope of the line between origin and point of static friction in the force-distance diagrams (figures 4*d* and 5*d*).

4.2 $$F_{c}\propto \sqrt{G_{c}}\sqrt{\frac{A}{C}}=\sqrt{G_{c}}\sqrt{AK},$$

where *G*_*c*_ is the critical strain energy release rate. This model has been applied successfully to explain the friction performance of a variety of synthetic and biological adhesive systems with different geometries and material properties on flat substrates [20,21,30,42,53]. For the adhesives with variable softness created in this study, we find a linear relation between friction and $\sqrt{AK}$ (figure 6), suggesting applicability of the GASL also on non-flat substrates such as the glass cylinder used here. Importantly, the GASL is valid only for catastrophic contact failure (i.e. abrupt detachment of the whole adhesive), and not for gradual peeling failure [54,55]. The linear scaling shown in figure 6 suggests applicability of the GASL and thus catastrophic failure. However, after reaching maximum friction we also found sliding and dynamic friction, which contradicts pure catastrophic failure. Future studies may elucidate the contact failure dynamics, from contact formation to failure, using continuous FTIR-based contact area imaging and tactile sensors (e.g. [56]). The latter approach also allows contact stress mapping, which may help explain the observed difference in average shear stress between adhesives with PUR-foam and hydroskeleton backings (figures 4*a*_4_ and 5*a*_4_). Future work may also investigate the effect of a soft backing on the friction of an adhesive in dependence of variations in adhesive surface design (e.g. gecko-inspired ‘hair-like’ structures compared to tree-frog-inspired ‘pillar-like’ structures), and of variations of substrate curvature relative to adhesive size.

The GASL predicts that the friction of a given adhesive system can be tuned through setting the product *AK*, i.e. maximizing the contact area as well as the adhesive’s tensile stiffness. Previous fibre-reinforced adhesives that tested this prediction were fabricated by embedding fabrics in thin layers of polydimethylsiloxane (PDMS; e.g. [30]), which allowed partial adjustment of the term *A K* through using different fabrics with variable stiffness *K*. This approach is non-dynamic as it requires refabrication of the whole system to vary *K*. Here, the friction performance of a single adhesive specimen with hydroskeleton backing was tuned during the contact formation phase by varying the applied internal pressure or opening the connection to a reservoir volume (figure 5*c*_1_,*c*_2_), demonstrating that friction performance on curved substrates can be dynamically controlled in a single adhesive without refabrication.

But was this friction tuning achieved by variation of contact area, compliance or both? To address this question, we simplify our experimental setting as laboratory shear experiment on an adhesive elastomer strip with height *h* and shear modulus *γ* mounted on an inextensible reinforcement layer, for which the GASL can be expressed as [55,57]

4.3 $$F_{c}=A\sqrt{\frac{G_{c}\gamma }{h}}.$$

The predicted linear proportionality between *F*_*c*_ and *A* matches our results well (figures 4*c*_1_ and 5*c*_1_). The obtained coefficients of determination (*R*^2^) of 0.91–0.92 show that more than 90% in force variation are explained by area variation. The remaining ∼10% in force variation may relate to manufacturing error, affecting the term $\sqrt{G_{c}\gamma h^{-1}}$ (e.g. variations in adhesive height *h*), and to measurement error (e.g. *F*_stat_ and *A*_stat_ were not measured at the exact same time point). This finding highlights the importance of a soft backing in dynamically tuning the friction of fibre-reinforced adhesives on non-flat substrates. Future work may investigate alternative methods to control the adhesive’s compressibility and tensile stiffness, for example by using materials with electroprogrammable stiffness [58], granular jamming [59–61] or various other methods [57,62].

In conclusion, we show that a bioinspired soft backing helps to form a large contact area and to create strong friction on non-flat substrates. For both tested backing variations, a positive relation was found between backing compressibility and created friction, which suggests generalizability of this mechanism to a wide range of adhesive designs. Moreover, the friction performance of the adhesives with bioinspired soft backings developed in this study can be tuned according to a generalized adhesion scaling law [20] even on non-flat substrates, further emphasizing the relevance of soft backings in the design of functional surfaces for adhesion and gripping technologies.

### 4.2. Soft backings in biological adhesives

Soft backings are also part of the adhesive organs of a wide range of terrestrial vertebrates such as amphibians (e.g. [35–37]), reptiles (e.g. [32,39]) and mammals like possums [63] and bats [64,65]. Various hypotheses have been proposed regarding the functional relevance of these structures. Russell hypothesized that the complex blood sinus in the toes of the Tokay gecko (*Gekko gecko*) stiffens upon compression, and thus helps to ‘conform to irregularities in the substratum’ [39] and to ‘provide an appropriate perpendicular preload’ [40] on the micropatterned ventral surface of the toes. Similar functionality has been suggested for the network of capillary blood vessels in the toes of the Australian green tree frog (*Litoria caerulea*; [38]). Rosenberg & Rose [63] speculated—also in line with Russell’s work—that the blood sinuses found in the toes of several opossum species ‘may help transmit pressure to the apical pad’. Hill *et al.* [48] showed experimentally that tree frog toe pads can conform to relatively narrow cylinders, enabling these animals to climb strongly curved substrates.

Our synthetic mimics of biological fibre-reinforced adhesive organs with soft backings allow us to experimentally assess these—to our knowledge still untested—hypotheses on the functional relevance of a soft backing. Clearly, the created mimics are less complex in their structures and materials than their natural counterparts. However, given that adding a soft backing enhances an adhesive’s contact area and friction performance irrespective of the exact backing design (PUR-foam versus hydroskeleton), our data support the original hypothesis of Russell [39] that a soft backing increases the conformability of biological adhesives to non-flat substrates. That being said, it is likely that the soft backings found in biological adhesives are multifunctional, and additional hypothesized functions such as shock absorption [38] or providing structural stability [65] cannot be excluded.

Soft backings have also been found in the adhesive organs of invertebrates. For example, the tarsal pads of the bush cricket (*Tettigonia viridissima*; [66]) and of the Asian weaver ant (*Oecophylla smaragdina*; [67]) contain volumes of haemolymph. By contrast to vertebrate bioadhesion research, some simulation and experimental studies are available on the functional relevance of soft backings in insects. Importantly, these investigations focused on adhesion and not on the frictional contact studied here. Finite-element modelling of the contact mechanics of the fluid-filled adhesive pads of orthopterans showed a negative correlation between contact area (and thus adhesion) and pad stiffness due to easier flattening of softer curved pads when pressed onto a flat substrate [68]. Our data show a similar trend for the inverse situation (i.e. a flat adhesive being pressed onto a curved substrate), and the generation of friction. More recently, Dening *et al.* [69] and Afferante *et al.* [70] used an elastic membrane spanned over a cylinder with a piston on the other side as mimic of soft bioadhesive pads, and proposed pressure-based curvature variation as the mechanism of adhesion control in biological adhesives. This explanation of variable attachment force based on shape variation differs from our work, which suggests force control through variation in effective material properties. Further investigation—for example the dynamic characterization of material properties and geometry throughout a contact cycle—is required to clarify whether biological adhesives tune their attachment strength through variation in geometry, material properties or both.

Finally, this study raises the question if (and how) organisms are able to dynamically tune the compressive stiffness of their adhesive organs? This question has been treated only hypothetically so far. Dai & Gorb [68] speculated that orthopterans may be able to control the pressure in the air sacks in their adhesive pads through active ‘breath’. Active stiffness control has also been suggested for the adhesive feet of the Tokay gecko (contraction of smooth muscles around the blood sinus; [32]) and the tree frog *Hyla cinerea* (contraction of smooth muscle fibres traversing the lymph space; [22]). By contrast, Federle *et al.* [67] proposed a localized ‘passive’ pump mechanism in the adhesive pads of ants, possibly in combination with active haemolymph transport by the leg circulatory organs. Detailed studies are needed to investigate these hypothesized mechanisms of stiffness control. We propose as a general framework for such a study on a given adhesive system (i) an accurate three-dimensional characterization of the morphology of the adhesive organ (e.g. through micro-computer-tomography), (ii) studying the movement of contrast-stained liquid in its respective reservoir (e.g. [71]), (iii) correlating liquid volume changes to muscle activation patterns and (iv) measuring pad stiffness while artificially controlling muscle activation.

### 4.3. Integrative design of biomimetic systems

In the beginning of the 2000s, the discovery of ‘dry’ adhesion underlying the remarkable attachment of geckos [72,73] set off an avalanche of designs of synthetic adhesive designs bearing variations of gecko-inspired surface patterning (see [25,27,28,74], for some reviews) with enhanced conformability to nano- and microscopic substrate irregularities. However, up-scaling of these adhesives to contact areas of more than a square centimetre remained a considerable challenge [20]. A decade later, Bartlett *et al.* [20,30,42,53] proposed a new paradigm for the design of gecko-inspired adhesives with embedded fibre-reinforcement for contact stress homogenization, which ultimately enabled up-scaling of the adhesives. While effective on flat substrates, functionality of these fibre-reinforced adhesives on non-flat substrates remained understudied. In this work, we provided such fibre-reinforced adhesives with conformability to macroscopic substrate irregularities by adding a bioinspired soft backing, which represents a crucial next step in the design of biomimetic adhesives that ultimately should reach the functionality of their biological models.

The gradual progress in the design of bioinspired adhesives from patterned surfaces over internal fibre-reinforcements to soft backings illustrates the need for a more integrative approach in biomimetic design. Whereas a focus on an individual features such as surface patterning is indispensable to illuminate the fundamental mechanisms that underlie its functionality, only the holistic assessment of all relevant aspects of a biological model system (i.e. morphology, biomechanics, behaviour, ecology and phylogeny; [75,76]) will enable the design of biomimetic devices that rival their biological models and can be employed in the ‘real’ world. For example, experimental studies on stick insects [77] and tree frogs [48] indicate the importance of behavioural aspects in contact formation on curved substrates.

Multiple lines of inquiry may advance the biomimetic design of fibre-reinforced adhesives. For example, the adhesive toe pads of tree frogs contain more than one fibrous component through which the adhesive pad surface can be loaded [22]. The tree-frog-inspired implementation of multiple load pathways in future bioinspired adhesives may enable a more refined control of interfacial contact stresses and thus of attachment strength. Sophisticated attachment control presumably also relies on sensory input. Whereas human tactile reception has already inspired the design of synthetic sensory systems (e.g. [78,79]), the sensory physiology of the adhesive organs of geckos, tree frogs and other animals is virtually unexplored. Accordingly, more work is required to integrate sensing and control with the already explored surface patterning, fibre-reinforcement and soft backing of bioinspired adhesives. The here proposed fibre-reinforced adhesive with soft backing may serve as a blueprint for such efforts and ultimately for the design of functional robotic surfaces for real-world applications such as wall-climbing robots [80,81], soft agrorobotic grippers [82,83] and surgical tools [84,85].

## Data Availability

The data are provided in electronic supplementary material [86].

## Appendix A

**A.1. Contact area modelling**

The area of contact *A* between a rigid cylinder (i.e. the substrate) and an elastic halfspace (i.e. the adhesive) under a compressive load approximates as a product of the known adhesive’s length *L* and the arc length *S* of the cylinder’s circular sector (electronic supplementary material, figure S1):

A 1 A=LS.

The arc length can take a maximal value of the adhesive’s width *W*, and computes as a product of the known cylinder radius *R* and the opening angle *θ* of the circular sector:

A 2 S=θR.

The opening angle is a function of the height *h* of the triangular portion of the circular sector (i.e. apothem):

A 3 $$\theta =2\arccos\frac{h}{R},$$

where *h* can be expressed as a function of the height *d* of arced portion of the circular sector (i.e. sagitta):

A 4 h=R−d.

The sagitta equals approximately the indentation depth between a rigid cylinder and an elastic halfspace as predicted by Hertz theory for elastic contact [50]:

A 5 $$d=\frac{4F}{\pi E^{*}L},$$

where *F* is the applied compressive load and *E** is the adhesive’s effective elastic modulus, which computes from elastic modulus *E* and Poisson’s ratio *ν* as *E** = *E* (1 − *ν*^2^)^−1^.

Combining and simplifying the above equations yields:

A 6 $$A=2LR\arccos\left(1-\frac{4F}{\pi E^{*}LR}\right).$$

**A. 2. Test results for data normality and homoscedasticity**

See electronic supplementary material, table S1.

**A.3. Multiple comparison results for differences in contact area and friction**

See electronic supplementary material, table S2 and figure S2.
